# Novel Association of HK1 with Glycated Hemoglobin in a Non-Diabetic Population: A Genome-Wide Evaluation of 14,618 Participants in the Women's Genome Health Study

**DOI:** 10.1371/journal.pgen.1000312

**Published:** 2008-12-19

**Authors:** Guillaume Paré, Daniel I. Chasman, Alexander N. Parker, David M. Nathan, Joseph P. Miletich, Robert Y. Zee, Paul M. Ridker

**Affiliations:** 1 Center for Cardiovascular Disease Prevention, Brigham and Women's Hospital, Harvard Medical School, Boston, Massachusetts, United States of America; 2 Donald W. Reynolds Center for Cardiovascular Research, Brigham and Women's Hospital, Harvard Medical School, Boston, Massachusetts, United States of America; 3 Amgen, Inc., Cambridge, Massachusetts, United States of America; 4 Diabetes Center, Massachusetts General Hospital, Harvard Medical School, Boston, Massachusetts, United States of America; University of Alabama at Birmingham, United States of America

## Abstract

Type 2 diabetes is a leading cause of morbidity and mortality. While genetic variants have been found to influence the risk of type 2 diabetes mellitus, relatively few studies have focused on genes associated with glycated hemoglobin, an index of the mean blood glucose concentration of the preceding 8–12 weeks. Epidemiologic studies and randomized clinical trials have documented the relationship between glycated hemoglobin levels and the development of long-term complications in diabetes; moreover, higher glycated hemoglobin levels in the subdiabetic range have been shown to predict type 2 diabetes risk and cardiovascular disease. To examine the common genetic determinants of glycated hemoglobin levels, we performed a genome-wide association study that evaluated 337,343 SNPs in 14,618 apparently healthy Caucasian women. The results show that glycated hemoglobin levels are associated with genetic variation at the GCK (rs730497; P = 2.8×10^−12^), SLC30A8 (rs13266634; P = 9.8×10^−8^), G6PC2 (rs1402837; P = 6.8×10^−10^), and HK1 (rs7072268; P = 6.4×10^−9^) loci. While associations at the GCK, SLC30A8, and G6PC2 loci are confirmatory, the findings at HK1 are novel. We were able to replicate this novel association in an independent validation sample of 455 additional non-diabetic men and women. HK1 encodes the enzyme hexokinase, the first step in glycolysis and a likely candidate for the control of glucose metabolism. This observed genetic association between glycated hemoglobin levels and HK1 polymorphisms paves the way for further studies of the role of HK1 in hemoglobin glycation, glucose metabolism, and diabetes.

## Author Summary

Type 2 diabetes is a leading cause of morbidity and mortality in both the developed and developing world. Because the main metabolic characteristic of diabetes is increased blood glucose concentration, we sought to uncover the genetic determinants of glycated hemoglobin, an index of the mean blood glucose concentration of the preceding 8–12 weeks. Taking advantage of new technologies allowing us to interrogate genetic variation on a whole-genome basis, we found that variations in the GCK, SLC30A8, G6PC2, and HK1 genes are important determinants of glycated hemoglobin concentrations. While associations with the GCK, SLC30A8, and G6PC2 genes have previously been identified in genetic studies of diabetes and blood glucose concentration, the findings at HK1 are novel. HK1 encodes the enzyme hexokinase, responsible for the first step in glucose utilization and a likely candidate for the control of glucose metabolism. This observed genetic association between glycated hemoglobin levels and HK1 genetic variants paves the way for further studies of the role of HK1 in glucose metabolism and diabetes.

## Introduction

Type 2 diabetes is a leading cause of morbidity and mortality. While genetic variants influence the risk of type 2 diabetes mellitus [1], relatively little is known about the role of genetic variations in the regulation of glucose concentration in healthy individuals. Indeed, only two genes, glucokinase (GCK) [2]–[4] and G6PC2 [5],[6], have been unequivocally associated with fasting blood glucose concentration in healthy subjects. Other genes, such as SLC30A8, have been linked to glucose metabolism without ever having been associated with blood glucose concentration per se [7].

Glycated proteins are formed post-translationally from the slow, non-enzymatic attachment of glucose to N-terminal valine and internal lysine amino groups [8]. The concentration of glycated hemoglobin is a function of the concentration of glucose to which the erythrocytes are exposed over their lifespan (120 days on average). The glycated hemoglobin assay reflects the integrated blood glucose concentration of the preceding 8–12 weeks [9], provides a better estimate of mean glycemia than routine determinations of blood glucose concentration, and is the most widely used index of chronic glycemia [10],[11]. Clinical trials have documented the relationship between glycated hemoglobin levels and the development of long-term complications in type 1 and type 2 diabetes [12],[13], and higher glycated hemoglobin levels in the subdiabetic range have been shown to predict type 2 diabetes risk [14] and cardiovascular disease [14]–[17].

Despite considerable effort to decipher the genetic basis of type 2 diabetes, relatively few studies have focused on the genetic basis of glycated hemoglobin levels. To examine the common genetic determinants of glycated hemoglobin levels, we performed a genome-wide association study evaluating 337,343 SNPs in 14,618 apparently healthy non-diabetic women of Caucasian ancestry.

## Materials and Methods

### WGHS Study Participants

The study population derived from the Women's Genome Health Study (WGHS) [18]. Briefly, participants in the WGHS include women from the United States Women's Health Study (WHS) with no prior history of cardiovascular disease, cancer, or other major chronic illness [19]. All participants provided a blood sample at the time of study enrollment from which genomic DNA was extracted.

For all WGHS participants, baseline glycated hemoglobin (HbA1c) was measured using the Tina-Quant turbidimetric inhibition immunoassay (Roche Diagnostics, Indianapolis, Ind) on a Hitachi 911 autoanalyzer using packed red blood cells. This assay is standardized against the approved International Federation of Clinical Chemists reference method and has a coefficient of variation of 7.2%. In addition to excluding women with self-reported diabetes at baseline, individuals with glycated hemoglobin of 7.0% or more were removed from analysis as this level has been proposed as diagnostic of drug-requiring diabetes [20]. The study was approved by the institutional review board of the Brigham and Women's Hospital.

### Genotyping

DNA samples were genotyped with the Infinium II technology from Illumina. Either the HumanHap300 Duo-Plus chip or the combination of the HumanHap300 Duo and I-Select chips was used. In either case, the custom content was identical and consisted of candidate SNPs chosen without regard to allele frequency to increase coverage of genetic variation with impact on biological function including metabolism, inflammation or cardiovascular diseases. Genotyping at 318,237 HumanHap300 Duo SNPs and 45,571 custom content SNPs was attempted, for a total of 363,808 SNPs. Genetic context for all annotations are derived from human genome build 36.1 and dbSNP build 126.

SNPs with call rates <90% were excluded from further analysis. Likewise, all samples with percentage of missing genotypes higher than 2% were removed (N = 1426). No obvious bias distinguished failed from successful samples and failures are therefore assumed to be the result of random technical artifacts. Among retained samples, SNPs were evaluated for deviation from Hardy-Weinberg equilibrium using an exact method [21] and were excluded when the P-value was lower than 10^−6^. Samples were further validated by comparison of genotypes at 44 SNPs that had been previously ascertained using alternative technologies. SNPs with minor allele frequency >1% in Caucasians were used for analysis. After quality control, 308,625 HumanHap300 Duo SNPs and 28,718 custom content SNPs were left, for a total of 337,343 SNPs.

### Population Stratification

Because population stratification can result in inflated type I error in genome-wide association analysis, a principal component analysis using 1443 ancestry informative SNPs was performed using PLINK [22] in order to confirm self-reported ancestry. Briefly, these SNPs were chosen based on Fst>0.4 in HapMap populations (YRB, CEU, CHB+JPT) and inter-SNP distance at least 500 kb in order to minimize linkage disequilibrium. Different ethnic groups were clearly distinguished with the two first components. Based on this analysis, 58 individuals were excluded from further evaluation as they did not cluster with other Caucasians, leaving 14,618 participants for the current study population. Two additional steps were taken to rule out the possibility that residual stratification within Caucasians was responsible for the associations observed. First, association analysis was done with correction by genomic control. Second, a principal component analysis [23] was performed in previously identified Caucasians (only) using 124,931 SNPs chosen to have pair-wise linkage disequilibrium lower than r^2^ = 0.4. The first ten components were then used as covariates in the association analysis. As adjustment by these covariates did not change the conclusions, we present analysis among Caucasian participants without further correction for sub-Caucasian ancestry. The value of the genomic control was 1.03916 before adjustment for the first ten components and 1.03328 after adjustment.

### Association Analysis

To identify common genetic variants influencing glycated hemoglobin levels, we first attempted to discover which loci significantly contributed to glycated hemoglobin concentration. Glycated hemoglobin concentrations were adjusted for age, menopause and body mass index using a linear regression model in R to reduce the impact of clinical covariates on glycated hemoglobin variance. The normal distribution of adjusted glycated hemoglobin values was checked by visual inspection of the normal Quantile-Quantile plot. Adjusted glycated hemoglobin values were then tested for association with SNP genotypes by linear regression in PLINK [22], assuming an additive contribution of each minor allele [24]. Beta coefficients are given using the major allele as the reference allele (major and minor alleles are presented in Supplementary Table S2). We also tested for association using genotypic, recessive and dominant genetic models. Since no additional locus was identified using these models we only present the results of the additive genetic model. A conservative P-value cut-off of 5×10^−8^ was used to correct for the maximum of 1,000,000 independent statistical tests thought to correspond to all the common genetic variation of the human genome [25]. The false discovery rate was also calculated as described by Benjamini and Hochberg [26].

Once any locus with genome-wide significance was identified, a forward selection linear multiple regression model was used to further define the extent of the genetic association. Briefly, all genotyped SNPs within 100 kb of the most significantly associated SNP at each locus and passing quality control requirements were tested for possible incorporation into a multiple regression model. In stepwise fashion, a SNP was added to the model if it had the smallest P-value among all the SNPs not yet included in the model and if it was statistically significant after adjusting for multiple comparisons. SNPs selected by this algorithm were also used in haplotype analysis using WHAP [27], as implemented in PLINK [22].

### Validation Sample

We sought to validate any novel findings from the WGHS in an independent replication sample comprised of 455 non-diabetic Caucasian participants recruited from the Boston metropolitan area [28]. A history of diabetes, or use of anti-diabetic medications, and/or fasting glucose levels >125 mg/dL, or abnormal oral glucose tolerance test results were used to exclude persons with diabetes in this cohort. Glycated hemoglobin was measured with an HPLC assay that serves as a primary reference assay for the National Glycohemoglobin Standardization Program, with coefficients of variation <2.5% for low and high standards. Genotype determination was performed with an ABI fluorescence-based allelic discrimination method (rs7072268) [29] or a single reaction kinetic thermal cycling method (rs2305198) [30]. The study was approved by the Brigham and Women's Hospital and Massachusetts General Hospital Institutional Review Boards for human subjects research.

## Results

**Figure 1:**
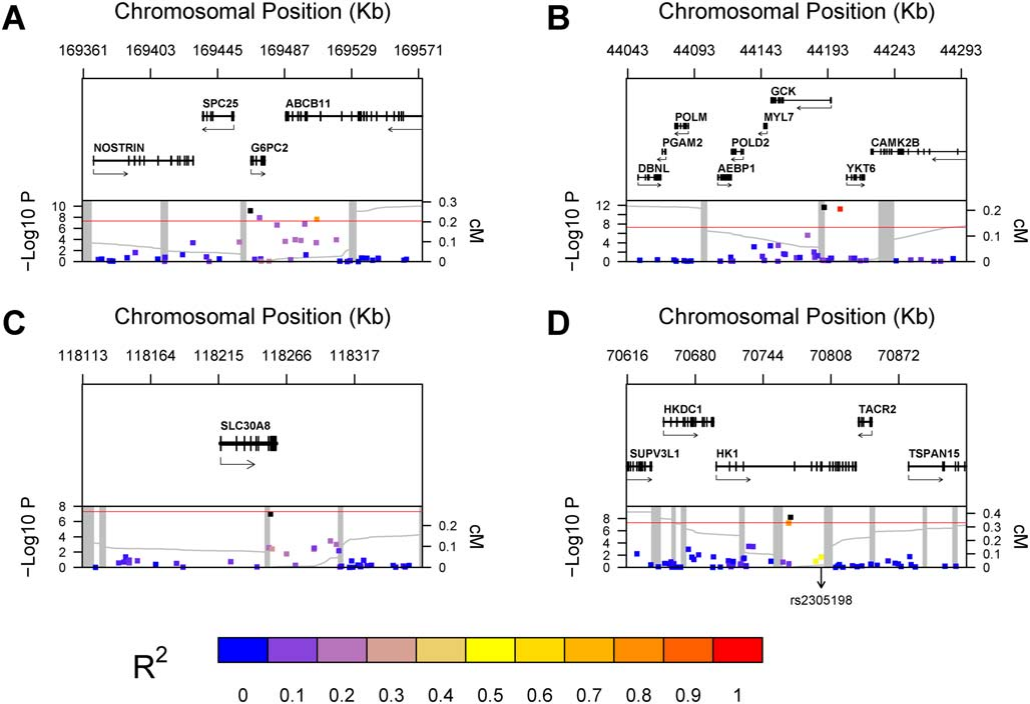
Genetic Context of Significant Associations. Genomic context for each of four loci with significant association with glycated hemoglobin levels. (A) G6PC2 locus (2q24.3); (B) GCK locus (19p13.2); (C) SLC30A8 locus (8q24.11); (D) HK1 locus (10q21.3). Upper panel: Genes from RefSeq release 25. Only one isoform is shown when multiple splicing variants are known. Lower Panel: SNPs are shown according to their physical location and −log_10_ P-values for association with glycated hemoglobin. At each locus, the SNP with the lowest P-value is represented by a black dot. The color of each other SNP was determined according to its linkage disequilibrium (r^2^) with the SNP with the lowest P-value, with colors varying from blue (r^2^ = 0) to red (r^2^ = 1). In Figure 1-D, the chromosomal position of rs2305198 is shown as this SNP was selected by the model selection algorithm in addition to the SNP with the lowest P-value (see text for details). The red line represents the genome-wide significance threshold of 5×10^−8^. Also shown is the genetic distance in cM from the lowest P-value SNP (light grey line) along with the position of recombination hotspots (light grey vertical bars). Recombination rates and hotspots are based on HapMap data, as described by McVean et al.[46] and Winckler et al.[47].

Clinical characteristics of the 14,618 non-diabetic women in the primary WGHS analysis and of the 455 non-diabetic men and women in the external validation cohort are provided in Supplementary Table S1. Results of the genome-wide association analysis of adjusted baseline glycated hemoglobin concentration are presented in Table 1, which includes all SNPs with P-value lower than 10^−6^. Six SNPs at three different loci – G6PC2, GCK and HK1 - had an association P-value lower than our previously defined genome-wide significance threshold of 5×10^−8^: rs1402837, rs560887 and rs6709087 at G6PC2; rs730497 and rs4607517 at GCK; rs7072268 at HK1. In addition, two SNPs, one at the HK1 locus and one at the SCL30A8 locus, had association P-values just above this threshold level (5.5×10^−8^ and 9.8×10^−8^, respectively). Genetic context of all these loci is presented in Figure 1 along with the −log_10_ transformed P-values.

**Table 1:** SNPs with a P-Value Lower than 10^−6^ for Association with Adjusted Glycated Hemoglobin.

SNP	Locus	Position (kb)	Nearest Gene	Function	MAF^a^	HW^b^	Beta (%HbA1c)^c^	P-Value^c^	FDR^e^
rs1402837	2q24.3	169466	G6PC2	-	0.23	0.87	0.023	6.8E-10	7.8E-05
rs560887	2q24.3	169471	G6PC2	intron	0.29	0.88	−0.020	1.2E-08	8.1E-04
rs563694	2q24.3	169482	G6PC2	-	0.34	0.76	−0.017	2.5E-07	7.8E-03
rs552976	2q24.3	169500	G6PC2	intron	0.35	0.97	−0.018	1.7E-07	5.8E-03
rs6709087	2q24.3	169507	G6PC2	intron	0.23	0.68	0.021	2.3E-08	1.3E-03
rs730497	7p13	44190	GCK	intron	0.17	0.82	0.030	2.8E-12	9.6E-07
rs4607517	7p13	44202	GCK	-	0.17	0.67	0.029	6.2E-12	1.1E-06
rs13266634	8q24.11	118254	SLC30A8	NSC^d^	0.30	0.50	−0.019	9.8E-08	3.8E-03
rs906216	10q21.3	70768	HK1	intron	0.44	0.15	−0.017	5.5E-08	2.7E-03
rs7072268	10q21.3	70770	HK1	intron	0.50	0.10	0.018	6.4E-09	5.5E-04

a MAF: Minor Allele Frequency.

b HW: Deviation from Hardy-Weinberg equilibrium P-value.

c All analyses were performed using adjusted glycated hemoglobin values (see text for details).

d NSC: Non-Synonymous Coding SNP.

e FDR: False Discovery Rate.

**Figure 2:**
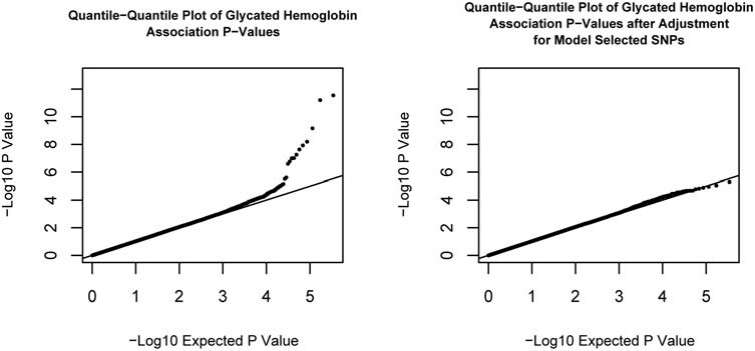
Quantile-Quantile plot of Glycated Hemoglobin Association P-Values. The quantile-quantile plot of glycated hemoglobin association P-values is shown on the left. On the right, the same quantile-quantile plot is shown, but after adjusting glycated hemoglobin values for the additive genetic effect of the 5 SNPs retained by the model selection algorithm (rs1402837, rs730497, rs13266634, rs7072268 and rs2305198; see text for details).

To define further the extent of genetic associations at these 4 loci, we applied a forward model selection algorithm to each of them, identifying SNPs that were non-redundantly associated with glycated hemoglobin. Briefly, 38 SNPs at GCK, 31 at SLC30A8, 41 at HK1 and 44 at G6PC2 were initially assessed for possible inclusion in a multiple linear regression model. Using a P-value cut-off of 3×10^−4^ to account for the 154 SNPs considered, 5 SNPs were selected, representing the lead SNP at each of the 4 loci considered plus one SNP (rs2305198; located at 70799 Kb on chromosome 10) at the HK1 locus (see Table 2). Interestingly, this later SNP (rs2305198; HK1) was marginally significant in univariable analysis (p = 0.02), illustrating that its inclusion in the model and significant association are conditional on the genotypes at rs7072268 (HK1). Multiple regression beta coefficients and P-values of the 5 SNPs selected are shown in Table 2 and range from 5.4×10^−8^ to 1.8×10^−25^. Shown in Figure 2 are the quantile-quantile plots of association P-values before and after adjusting glycated hemoglobin concentration for the combined effect of these 5 SNPs. Among these SNPs, there was no strong evidence for non-additive effects of the minor allele as judged by lack of significance for a likelihood ratio test comparing the additive regression model to an alternative genotype model with an additional degree of freedom. No pairwise gene-gene interaction was observed between any of the model selected SNPs. That is, we tested every possible 2-SNPs combination among these 5 SNPs.

**Table 2:** Multiple Linear Regression Statistics of SNPs Retained by the Forward Model Selection Algorithm.

Locus	SNP	Nearest Gene	Function	MAF^a^	HW^b^	Beta^c^	P-Value^c^
2q24.3	rs1402837	G6PC2	-	0.23	0.87	0.0236	4.7E-10
7p13	rs730497	GCK	intron	0.17	0.82	0.0291	5.7E-12
8q24.11	rs13266634	SLC30A8	NS^d^	0.30	0.50	−0.0189	5.4E-08
10q21.3	rs7072268	HK1	intron	0.50	0.10	0.0465	1.8E-25
10q21.3	rs2305198	HK1	intron	0.38	0.05	0.0409	4.3E-19

a MAF: Minor Allele Frequency.

b HW: Deviation from Hardy-Weinberg equilibrium P-value.

c All analyses were performed using adjusted glycated hemoglobin values (see text for details). Beta coefficients and P-values were derived form a multiple linear model that included all 5 SNPs.

d NS: Non-Synonymous Coding SNP.

We sought to replicate the novel association between glycated hemoglobin level and HK1 SNPs (rs2305198 and rs7072268) in the external validation sample of 455 non-diabetic men and women from the Boston metropolitan area. We included both SNPs (assuming an additive genetic effect) in a multiple regression model that also included age, sex, menopause and BMI as covariates. Both SNPs were successfully replicated, with two-sided P-values of 0.027 (rs2305198) and 0.050 (rs7072268), and both with consistent direction of effect. Furthermore, the effect size (i.e. beta coefficients) was larger than in the WGHS, with changes in glycated hemoglobin of 0.101% (rs2305198) and 0.085% (rs7072268) per allele in the replication sample, as opposed to 0.041% and 0.046% respectively in the WGHS. The association was validated in both men (N = 204) and women (N = 251), without any evidence of interaction with sex (data not shown). Both SNPs were in Hardy-Weinberg equilibrium, had allelic frequencies consistent with the ones observed in WGHS, and were successfully genotyped in over 93% of individuals. Of note, the P-values for rs2305198 and rs7072268 were non-significant (p>0.05) when each SNP was analyzed separately in a linear model. Overall, the P-values for rs2305198 and rs7072268 when both SNPs were included in a multiple regression model that used the combined WGHS and replication sample were 9.9×10^−20^ and 1.7×10^−25^, respectively.

The 2 SNPs at the HK1 locus selected by our algorithm were also used in haplotype analysis in WGHS individuals (Table 3). The estimate of the proportion of variance attributable to haplotypes, as well as their regression coefficients, is consistent with the linear model of these same SNPs, reinforcing the adequacy of the multiple linear model to explain the association. Linkage disequilibrium between these two HK1 SNPs was 0.49 for r^2^ and 0.90 for D'. The 2 SNPs at HK1 collectively explained 0.7% of the total variance in glycated hemoglobin concentration, whereas the G6PC2 SNP (rs1402837) explained 0.2%, the GCK SNP (rs730497) 0.3% and the SLC30A8 SNP (rs13266634) 0.2%. In comparison, clinical covariates accounted for 9.5% of the variance (Table 4), and together the candidate loci and the clinical variables accounted for 10.9% of total variance. As was the case in the WGHS, haplotype analysis of the replication sample yielded similar results as the multivariable linear regression, albeit with lesser statistical significance (data not shown).

**Table 3:** Haplotype Analysis of rs7072268 and rs2305198 (HK1 locus) in Relation to Adjusted Glycated Hemoglobin.

Haplotype		Frequency	Beta (%HbA1c)	P-Value
rs7072268	rs2305198			
A	G	0.02	0.033	0.012
G	G	0.36	0.006	0.095
A	A	0.48	0.017	2.2E-07
G	A	0.14	−0.049	1.5E-26

The omnibus (3 df) p-value from the joint haplotype analysis is 2.1E-25.

**Table 4:** Partition of Glycated Hemoglobin Variance According to Genetic and Clinical Variables.

Category	Variable	Variable R^2^	Category R^2^
Clinical Covariates	Age	0.0354	0.0946
	Body Mass Index	0.0585	
	Menopause Status	0.0006	
2q24.3 (G6PC2) Locus	rs1402837	0.0024	0.0024
7p13 (GCK) Locus	rs730497	0.0029	0.0029
8q24 (SLC30A8) Locus	rs13266634	0.0019	0.0019
10q21.3 (HK1) Locus	rs7072268	0.0021	0.0070
	rs2305198	0.0049	
TOTAL			0.1088

Since several SNPs have been unequivocally associated with type 2 diabetes, we tested whether these “candidate” SNPs [1],[4],[31],[32] were associated with glycated hemoglobin as well (Table 5). When a SNP was not directly typed in WGHS, we used HapMap CEU linkage disequilibrium data to find the WGHS SNP with the highest r^2^ with that SNP. While all SNPs tested had a direction of association consistent with their effect on the risk of diabetes, only 10 SNPs had a (one-sided) P-value lower than 0.05 (Table 5). Overall, the one-sided association P-value for the 19 SNPs taken together (i.e. the P-value obtained by combining all 19 one-sided P-values; this was done using Fisher's method for combining P-values from independent tests) was 3.0×10^−22^. The P-value was 8.5×10^−9^ when the SLC30A8 SNP rs13266634 and the GCK SNP rs730497 were removed. It has previously been shown that the metabolic effects of rs8050136 (FTO locus) are mediated mainly through increased body weight [33],[34]. Adjustment of glycated hemoglobin for BMI would therefore be expected to dampen the association of rs8050136 (FTO locus) with glycated hemoglobin level. Indeed, non-adjusted glycated hemoglobin showed stronger association with rs8051136 (p = 0.007).

**Table 5:** Association of Glycated Hemoglobin with Type II Diabetes Candidate SNPs.

Gene(s)	Candidate SNP	Best WGHS Proxy	r^2^	D'	Beta (%HbA1c)	P-Value (one-sided)	Direction of Effect in Agreement
ADAMTS9	rs4607103	rs4132228	0.78	1	−0.004	0.11	YES
CDC123,CAMK1D	rs12779790	rs11257622	0.79	0.95	0.006	0.07	YES
CDKAL1	rs10946398	rs4712523	1	1	0.004	0.11	YES
CDKN2A-2B	rs10811661	rs2383208	1	1	−0.008	**0.02**	YES
FTO	rs8050136	rs8050136	1	1	0.003	0.18	YES
GCK	rs1799884	rs730497	1	1	0.030	**1.4E-12**	YES
GCKR	rs780094	rs780094	1	1	−0.007	**0.01**	YES
HHEX-IDE	rs1111875	rs1111875	1	1	−0.008	**4.8E-03**	YES
IGF2BP2	rs4402960	rs1470579	1	1	0.008	**0.01**	YES
JAZF1	rs864745	rs1635852	0.97	1	−0.001	0.34	YES
KCNJ11	rs5215	rs5215	1	1	0.002	0.28	YES
NOTCH2	rs10923931	rs2793831	1	1	0.001	0.44	YES
PPARG	rs1801282	rs1899951	1	1	−0.008	**0.05**	YES
SLC30A8	rs13266634	rs13266634	1	1	−0.019	**4.9E-08**	YES
TCF2	rs4430796	rs7501939	0.76	1	0.007	**0.02**	YES
TCF7L2	rs7901695	rs7903146	0.80	0.95	0.009	**3.5E-03**	YES
THADA	rs7578597	rs6708660	0.16	1	−0.007	**0.01**	YES
TSPAN8,LGR5	rs7961581	rs1353362	0.96	1	0.005	0.10	YES
WFS1	rs10010131	rs10012946	1	1	−0.004	0.12	YES

All analyses were performed using adjusted glycated hemoglobin values (see text for details).

Linkage disequilibrium between candidate SNPs and their best WGHS proxies (r^2^ and D') were derived from HapMap data build 35 (CEU individuals).

One-sided P-values equal or lower than 0.05 are shown in bold.

## Discussion

Four loci – GCK, SLC30A8, HK1 and G6PC2 – have been identified in this report as being significantly associated with glycated hemoglobin levels in non-diabetic individuals. While genetic variants of GCK [2]–[4] and G6PC2 [5],[6] are known to influence fasting glucose concentrations in non-diabetics, the association with the diabetes gene SLC30A8 [7] has not been previously identified. In contrast, genetic associations with glycated hemoglobin have previously been described with the candidate genes calpain-10 (CAPN10) [35], resistin (RETN) and adiponectin (ADIPOQ) loci [36], although none of these associations were replicated in our data.

The most striking association observed in the current report involves HK1, which is a novel finding. Four different hexokinase isozymes, named type I, II, III and glucokinase (GCK), are present in mammalian tissues. Hexokinase type I (HK1) is the predominant isoform found in erythrocytes, although it is also expressed in other human tissues that depend strongly on glucose utilization for their physiologic functioning, such as brain and muscle [37],[38]. Despite this wide tissue distribution, the main clinical manifestation of rare HK1 mutations is severe nonspherocytic hemolytic anemia [38]. To our knowledge, no common genetic variation has previously been correlated with any phenotype. The reason why erythrocytes are particularly susceptible to these rare mutations is not clear, but the prevailing assumption is that tissues strongly dependent on HK1, such as erythrocytes, might be preferably affected. One hypothesis to explain the strong association of rs7072268 and rs2305198 with glycated hemoglobin levels is that these SNPs affect hexokinase concentration or activity in erythrocytes (or act as proxies for other HK1 functional variants). Since intra-erythrocyte glucose concentration is thought to be the main determinant of hemoglobin glycation rate [39] and hexokinase is the initial and rate-limiting enzymatic step in erythrocyte glucose metabolism, after glucose transporter mediated diffusion, it is likely that variations in HK1 activity would affect glycation. Alternatively, genetic variations at HK1 might modulate systemic glucose metabolism, raising the possibility that these variations are associated with the risk of incident diabetes. Indeed, HK1 is expressed in muscle, an insulin-sensitive tissue recognized for its importance in glucose metabolism.

SLC30A8 functions as a zinc transporter in secretory vesicles of pancreatic beta cells, providing zinc for insulin maturation and storage [40],[41]. A coding non-synonymous polymorphism of SLC30A8 (R325W; rs13266634) has been demonstrated to be protective for type 2 diabetes [32], [42]–[45] and was shown to influence insulin secretion following an intravenous glucose challenge [7]. In accordance with these results, that same coding SNP was associated with lower glycated hemoglobin in our study.

Because many genetic variants have been previously associated with the risk of type 2 diabetes, we also tested these for association with glycated hemoglobin. Although not genome-wide significant, all of these associations had a consistent direction of effect (as compared with their association with diabetes). The low combined P-value shows that it is very unlikely that these glycated hemoglobin associations had such a consistent direction of effect by chance alone, suggesting that they likely reflect underlying “true” associations. Furthermore, it also suggests that if we had analyzed a much bigger sample, some of these associations might have become genome-wide significant. To address the possibility that the very low P-value from the analysis of all 19 SNPs stems solely from the inclusion of the very significant SLC30A8 and GCK SNPs, we repeated the procedure using only the weakly associated SNPs as a sensitivity analysis, with similar results. It therefore appears that genetic variations affecting the risk of future diabetes do not necessarily have a marked impact on glycated hemoglobin in healthy women, pointing to different, yet partially overlapping, genetic architectures for glycated hemoglobin and diabetes.

The limitations of this study include less than certain elimination of diabetic subjects in the WGHS cohort, since we did not perform oral glucose tolerance testing. Therefore, some subjects with undiagnosed diabetes might have been included in that population. However, we believe it is unlikely this would have affected the results for two reasons. First, when we reproduced our analysis using only women with glycated hemoglobin lower than 6.0%, we obtained similar results. Second, we were able to replicate our novel HK1 findings in the validation cohort in which OGTT was used to eliminate diabetic participants. Although HK1 polymorphisms are associated with glycated hemoglobin levels, the functional consequences of these polymorphisms needs to be established.

In this report, we demonstrate that glycated hemoglobin levels in non-diabetic women are affected by genetic variation at the GCK, SLC30A8, G6PC2 and HK1 loci. While associations at the GCK, SLC30A8 and G6PC2 loci confirm or extend previous work done on the genetic basis of diabetes or fasting glucose, our replicated findings at HK1 are novel. It will be of considerable interest to study HK1 in non-Caucasian populations, as well as in pre-diabetic and diabetic populations, although the confounding effects of treatment might obscure any role of these polymorphisms in the diabetic population. This hypothesis generating genetic observation paves the way for further studies of the role of HK1 in hemoglobin glycation, glucose metabolism and diabetes. Whether the differences in hemoglobin glycation associated with genetic polymorphisms demonstrated herein are paralleled by differences in glycation in other tissues that are thought to underlie long-term complications of diabetes remains an open question.

## Supporting Information

Table S1 Clinical Characteristics of the Samples Used.
(0.04 MB DOC)

Table S2 Minor and Major Alleles.
(0.04 MB DOC)
