# Supplementary material for: Novel Association of HK1 with Glycated Hemoglobin in a Non-Diabetic Population: A Genome-Wide Evaluation of 14,618 Participants in the Women's Genome Health Study
Source: PLoS Genet. 2008 Dec 19;4(12):e1000312. doi: 10.1371/journal.pgen.1000312 (PMC2596965; doi:10.1371/journal.pgen.1000312)
Supplement: Table S1 — Clinical Characteristics of the Samples Used. (0.04 MB DOC) [file pgen.1000312.s001.doc]

**SUPPLEMENTAL DATA**

**Supplementary Table S1:** Clinical Characteristics of the Samples Used.

|  | **WGHS** | **Validation Sample** |
| --- | --- | --- |
|  | **(n=14,618)** | **(n=455)** |
| %Women | 100% | 55.2% |
| Age (yrs.) | 54.7 (7) | 51.8 (13.0) |
| BMI (kg/m^2) | 25.8 (4.8) | 26.7 (6.0) |
| Menopause^a^ | 54.7% | 51.8% |
| HRT^b^ | 44.8% | 15.5% |
| Smoking | 11.8% | 14.5% |
| C-Reactive Protein (mg/L) | 3.5 (5.3) | NA |
| Triglycerides (mg/dL) | 142.4 (88.8) | NA |
| HDL cholesterol (mg/dL) | 54.7 (15.4) | NA |
| HbA1c (%) | 5.0 (0.3) | 5.5 (0.5) |
| Results are given as mean (standard deviation), as appropriate. | | |
| ^a^In women only. | | |
| ^b^HRT: Hormone replacement therapy use, in women only. | | |
