# Supplementary material for: Novel Association of HK1 with Glycated Hemoglobin in a Non-Diabetic Population: A Genome-Wide Evaluation of 14,618 Participants in the Women's Genome Health Study
Source: PLoS Genet. 2008 Dec 19;4(12):e1000312. doi: 10.1371/journal.pgen.1000312 (PMC2596965; doi:10.1371/journal.pgen.1000312)
Supplement: Table S2 — Minor and Major Alleles. (0.04 MB DOC) [file pgen.1000312.s002.doc]

**SUPPLEMENTAL DATA**

**Supplementary Table S2:** Minor and Major Alleles

|  | **Major Allele** | **Minor Allele** |
| --- | --- | --- |
| rs13266634 | G | A |
| rs1402837 | G | A |
| rs2305198 | A | G |
| rs4607517 | G | A |
| rs552976 | G | A |
| rs560887 | G | A |
| rs563694 | A | C |
| rs6709087 | A | G |
| rs7072268 | G | A |
| rs730497 | G | A |
| rs906216 | C | A |
